# Risk factors for right colon, left colon and rectal cancers differ between men and women: the population‐based HUNT study in Norway

**DOI:** 10.1111/codi.16324

**Published:** 2022-09-13

**Authors:** Siv S. Brenne, Eivind Ness‐Jensen, Tom‐Harald Edna, Stian Lydersen, Eivor A. Laugsand

**Affiliations:** ^1^ Department of Internal Surgery, Levanger Hospital Nord‐Trøndelag Health Trust Levanger Norway; ^2^ Department of Public Health and Nursing, NTNU, HUNT Research Centre Norwegian University of Science and Technology Levanger Norway; ^3^ Department of Medicine, Levanger Hospital Nord‐Trøndelag Hospital Trust Levanger Norway; ^4^ Upper Gastrointestinal Surgery, Department of Molecular Medicine and Surgery, Karolinska Institutet Karolinska University Hospital Stockholm Sweden; ^5^ Department of Clinical and Molecular Medicine, NTNU, Norwegian University of Science and Technology Trondheim Norway; ^6^ Regional Centre for Child and Youth Mental Health and Child Welfare‐Central Norway, Faculty of Medicine and Health Sciences, NTNU Norwegian University of Science and Technology Trondheim Norway

**Keywords:** colorectal cancer, risk factors, sex disparities, tumour location

## Abstract

**Aim:**

The aim of this study was to assess established risk factors for colorectal cancer (CRC) separately for right colon, left colon and rectal cancer in men and women.

**Method:**

This was a prospective cohort study comparing incidental CRC cases and the general population participating in a longitudinal health study in Norway (the HUNT study).

**Results:**

Among 78 580 participants (36 825 men and 41 754 women), 1827 incidental CRCs were registered (931 men and 896 women). Among men, the risk of cancer at all locations increased with age [HR 1.46 (1.40–1.51), HR 1.32 (1.27–1.36), HR 1.30 (1.25–1.34) per 5 years for right colon, left colon and rectal cancer, respectively] and the risk of left colon cancer increased with higher body mass index [HR 1.28 (1.12–1.46) per 5 kg/m^2^]. The risk of right colon cancer (RCC) increased with smoking [HR 1.07 (1.04–1.10) per 5 pack years]. Among women, the risk of cancer at all locations increased with age [HR 1.38 (1.34–1.43), HR 1.23 (1.19–1.27), HR 1.20 (1.16–1.24) per 5 years] and smoking [HR 1.07 (1.02–1.12), HR 1.07 (1.02–1.12), HR 1.10 (1.05–1.17) per 5 pack years] for right colon, left colon and rectal cancer, respectively. The risk of RCC increased with night shift work [HR 1.93 (1.22–3.05)].

**Conclusion:**

The risk factors for developing CRC differ by anatomical location and sex. The relationship between risk factors and CRC may be more nuanced than previously known.

**What does this paper add to the literature?**

In colorectal cancer, screening, treatment and prophylactic protocols do not apply sex‐specific recommendations. This is the first large European study demonstrating that risk factors for colorectal cancer differ by sex and tumour location. Further knowledge could guide effective prevention policies and possibly reduce disease burden.

## INTRODUCTION

Colorectal cancer (CRC) has been considered as a single tumour entity. As colon cancer (CC) and rectal cancer (RC) differ in many ways, that is, macroscopically, histologically, molecularly, by survival, sensitivity to chemotherapy and effect of prophylactic measures, many now consider these as separate entities [1, 2]. Furthermore, CC may be divided into left colon cancer (LCC) and right colon cancer (RCC) based on the differences not only in anatomical location but also in the microbiome, clinical, chromosomal and molecular characteristics [3, 4, 5]. Women seem more prone to develop RCC and more aggressive forms of neoplasia, and they have higher mortality and lower 5‐year survival rates than men [4]. To date, most researchers have not considered tumour localization or sex disparities in their study design or interpretation [4]. Consequently, RCC, LCC and RC are well described as different entities, but little is known about the underlying risk factors separating the three, and how these differ in men and women.

The aetiology of CRC seems to be multifactorial. Established risk factors for CRC are increased age, male sex, diabetes mellitus, smoking, high alcohol consumption, obesity, high intake of red and processed meat, inflammatory bowel disease and family history of CRC or adenomatous polyps [6, 7, 8, 9, 10]. Physical activity, high intake of fish, dairy products, fruit, vegetables and fibres, as well as intake of medications such as hormone replacement therapy, acetylsalicylic acid, statins, certain vitamins, calcium or magnesium supplements may protect against the development of CRC [6, 10]. Night shift work, sleep duration, former treatment for testicular or prostate cancer, *Helicobacter pylori* infection and other infections are among the factors that have unclear effects on CRC risk [6, 11, 12, 13].

CRC is among the most preventable cancer types, as more than 50% of cases are attributable to lifestyle factors [6]. In addition, CRC is well suited for screening, as it reduces incidence and mortality as well as downstages the total tumour burden [14]. The prevalence of CRC in Norway is one of the world's highest, and for unknown reasons the incidence is ever‐increasing among both men and women [15]. A national screening programme has been decided upon but not implemented to date.

Screening guidelines in general do not apply sex‐specific recommendations, although it is well known that the various screening methods differ in their ability to detect RCC, LCC and RC among men and women [4]. Combining an efficient screening programme with sex‐specific lifestyle recommendations could reduce the CRC burden [14]. Hence, knowledge of how cancer risk factors vary with tumour localization and sex might be of importance to guide the health policy of CRC prevention. Therefore, our aim was to study the risk factors associated with RCC, LCC and RC, and whether these differ between men and women.

## METHOD

### Study design

**FIGURE 1:**
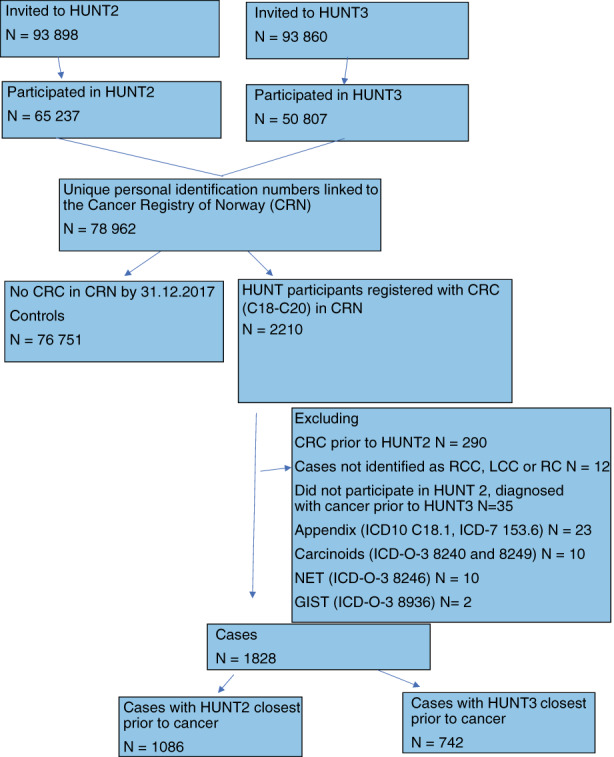
Flowchart of selection of cases and controls

This was a prospective cohort study based on the Trøndelag Health Study (HUNT). HUNT collected data from four surveys [HUNT1 (1984–6), HUNT2 (1995–7), HUNT3 (2006–8) and HUNT4 (2018–19)]. The entire adult population aged >20 years in Trøndelag County, Norway was invited to participate. The study included written questionnaires, standardized clinical measurements performed by trained personnel and blood samples, collected and stored in the HUNT Biobank. Through linkage to the Cancer Registry of Norway by the unique national personal identification number assigned to each Norwegian inhabitant, participants in HUNT2 and/or HUNT3 with RCC, LCC or RC were identified. The Cancer Registry of Norway has been responsible for the collection and organization of data from all institutions diagnosing and treating cancer patients in Norway since 1951. Reporting of these data is mandatory by law for all health personnel. A flowchart of the selection of the cases and controls is shown in Figure 1.

### Definition of outcome

The outcome of this study was the first CRC diagnosis among participants in HUNT2 and HUNT3 until 31 December 2017. CRC was identified according to the International Classification of Diseases, 7th edition (codes 153.0–154.0) and 10th edition [codes C18–20 (excluding C18.1 Appendix)] and the morphological codes according to the International Classification of Diseases for Oncology, 3rd edition (ICD‐O‐3) 8041, 8144, 8210–11, 8255–8263, 8480–8481, 8490, 8510, 8570–8574, 6900, 6999, 8000–8020 (excluding carcinoids 8240 and 8249, neuroendocrine carcinomas 8246 and gastrointestinal stromal tumours 8936). RCC was defined as ICD‐7 153.0–153.1, LCC was defined as ICD‐7 153.2–153.4 and RC was defined as ICD‐7 154.0. If synchronous cancers were registered (*n* = 51), and not located in the same anatomical subclassification (*n* = 7), the most distal cancer localization was used. The controls were defined as those participating in HUNT2 and/or HUNT3 who were not diagnosed with CRC in the Cancer Registry of Norway (1956 to 31 December 2017). For all participants, the cancer stage was scored based on data from hospital journals according to the tumour, node, metastasis (TNM) classification [16].

### Definition of exposures

A summary of known risk factors and their association with CRC, RCC, LCC and RC is presented in Table S1. Based on these studies, the following variables were selected as exposures: age, sex, body mass index (BMI), night shift work, diabetes mellitus, smoking, exercise, educational level, and intake of fruit/berries, vegetables, milk, bread and sausages/hamburgers. As there is a dose–response relationship between smoking and CRC risk, smoking was recorded as pack years [8]. Further definitions of the exposures are presented in Table 1.

**TABLE 1:** Definition of exposures

Risk factor	M/Q	Definition
BMI	M	Weight in kilograms divided by the squared height in metres [9]
Diabetes mellitus	Q	Answering ‘yes’ or ‘no’ to the question: ‘Do you or have you ever had diabetes?’
Smoking	Q	Pack years is packs of cigarettes [20] per day multiplied by the years smoked [8]
Fruit/berries, vegetables, processed meat^a^ and fish	Q	Answering ‘0–3 times per month’, ‘1–3 times per week’, ‘4–6 times per week’, ‘once a day’, ‘two or more times per day’ to the question: ‘How often do you normally eat this type of food?’
Milk	Q	Answering ‘never’, ‘<1 glass/day’, ‘1–3 glasses/day’ or ‘>3 glasses/day’ to the question: ‘How often do you drink milk?’
Bread	Q	Answering ‘white/white multigrain/semi wholegrain’ or ‘wholegrain/crispbread’ to the question: ‘What type of bread do you usually eat?’
Night shift work	Q	Answering ‘yes’ or ‘no’ to the question: ‘Do you work shifts, night work or on call?’
Exercise	Q	Answering ‘none’, ‘less than 1 hour/time per week’, ‘1–2 hours/time per week’ or ‘more than 3 hours/times per week’ to the question: ‘Over the last year, how often have you exercised light/hard?’ MET (metabolic equivalent) hours/week were calculated as in Rangul et al [18]
Education	Q	Answering ‘primary school 7–10 years, continuation school, folk high school’, ‘high school, intermediate school, vocational school, 1–2 years high school’, ‘university qualifying examination, junior college, A levels’, ‘university or other post‐secondary education, less than 4 years’ or ‘university/college, 4 years or more’ to the question: ‘What is your highest level of education?’

Abbreviations: BMI, body mass index; M, measurement; Q, questionnaire.

^a^ Hot dogs/sausages/hamburgers.

### Statistical analyses

The population characteristics are presented as frequencies, percentages, means and standard deviations. Of the two surveys, HUNT2 or HUNT3, the closest prior to the cancer diagnosis was chosen as baseline. The observational time from HUNT2 or HUNT3, respectively, was used as the time variable in survival analyses. CRC risk was assessed using Cox regression analysis. RCC, LCC and RC risk were assessed using competing risk analysis, applying the Fine and Grey method [17]. These analyses were carried out with one risk factor at a time, including the indicator variable of the baseline (HUNT2/3), the risk factor, sex, the interaction risk factor × sex, age, BMI and smoking. Available case analysis was used; that is, in each analysis, all subjects with data on the relevant variables were included. Hazard ratios (HRs) with 95% confidence intervals were reported separately for men and women, in addition to the *p*‐value for the interaction with sex. The predefined two‐sided significance level was set to 5%. However, due to multiple hypotheses, *p*‐values between 1% and 5% were interpreted with caution. The statistical software SPSS 26.0 was used for all statistical analyses (IBM Corp.). The Fine and Grey competing risk regression analyses were carried out using the SPSS extension command COMPRISK, which uses the R ‘cmprsk’ package [18].

### Ethics

All participants provided written informed consent when participating in HUNT, including consent for to linkage to their medical records as well as other central health registries in Norway. Confidentiality was strictly maintained during data storage and handling.

## RESULTS

### Study population

Among 78 579 participants (36 825 men and 41 754 women) in HUNT, 1828 (931 men and 897 women) were diagnosed with CRC. The remaining 76 751 participants were considered controls. The characteristics of patients and controls are presented in Table 2. The patients were generally older than the controls, and patients with RCC were older than LCC and RC cases. Among men, 351 cases were RCC, 317 LCC and 263 RC. Among women, 414 patients were RCC, 274 LCC and 209 RC. Most of the patients with RCC had Stage II–IV disease (81%), whereas most of the patients with RC had Stage I–II disease (52%).

**TABLE 2:** Characteristics of the study population

	Men, *n* = 36 825 (46.9%)					Women, *n* = 41 754 (53.1%)					
Total *N* = 78 579	CRC	Right colon	Left colon	Rectum	Controls	CRC	Right colon	Left colon	Rectum	Controls	Missing total
Cases *n* = 1828, controls *n* = 76 751	931 (2.5%)	351 (1%)	317 (0.8%)	263 (0.7%)	35894 (97.5%)	897 (2.1%)	414 (1%)	274 (0.6%)	209 (0.5%)	40 857 (97.9%)	
Age at inclusion (years)											0 (0%)
Mean (SD)	65.3 (11.6)	67.2 (11.2)	64.6 (11.7)	63.7 (11.5)	53.0 (17.4)	66.1 (11.9)	68.9 (10.6)	64.2 (12.5)	63.1 (12.4)	53.2 (18.1)	
Stage											126 (6.9%)
I	139 (14.9%)	24 (6.8%)	48 (15.1%)	67 (25.5%)	0 (0%)	140 (15.6%)	53 (12.8%)	39 (14.2%)	48 (23%)	0 (0%)	
II	312 (33.5%)	136 (38.7%)	101 (31.9%)	75 (28.5%)	0 (0%)	327 (36.5%)	146 (35.3%)	80 (29.2%)	54 (25.8%)	0 (0%)	
III	186 (20%)	69 (19.7%)	60 (18.9%)	57 (21.7%)	0 (0%)	201 (22.4%)	95 (23%)	58 (21.2%)	48 (23%)	0 (0%)	
IV	212 (22.8%)	93 (26.5%)	78 (24.6%)	41 (15.6%)	0 (0%)	185 (20.6%)	82 (19.8%)	63 (23%)	40 (19.1%)	0 (0%)	
Unknown	82 (8.8%)	29 (8.3%)	30 (9.5%)	23 (8.7%)	0 (0%)	44 (4.9%)	38 (9.1%)	34 (12.4%)	19 (9.1%)	0 (0%)	
Body mass index (kg/m^2^)											780 (1%)
>30.0	224 (24.2%)	86 (24.7%)	76 (24.1%)	62 (23.7%)	7101 (20%)	250 (28.2%)	114 (27.7%)	84 (31.3%)	52 (25.1%)	9292 (23%)	
Mean (SD)	27.6 (3.6)	27.6 (3.4)	27.7 (3.4)	27.4 (3.7)	27.1 (3.8)	27.7 (4.7)	27.7 (4.4)	28.2 (5.2)	27.1 (4.7)	26.8 (4.9)	
Diabetes mellitus											118 (0.2%)
Yes	65 (7%)	30 (8.6%)	20 (6.3%)	15 (5.7%)	1767 (4.9%)	60 (6.7%)	29 (7%)	18 (6.6%)	13 (6.2%)	1769 (4.3%)	
No	862 (93%)	319 (91.4%)	295 (93.7%)	248 (94.3%)	34 063 (95.1%)	833 (93.3%)	384 (93%)	253 (93.4%)	196 (93.8%)	39 042 (95.7%)	
Smoking											2600 (3.3%)
Pack years, mean (SD)	14.8 (16.0)	16.6 (16.8)	14.5 (16.4)	12.6 (14.1)	9.6 (13.6)	7.2 (11.1)	6.7 (11.4)	6.9 (9.9)	8.4 (12.2)	5.8 (9.3)	9996 (12.7%)
Fruit/berries											28 183 (35.9%)
0–3 times/month	17 (4.3%)	3 (2.1%)	8 (5.6%)	6 (5.3%)	1595 (7.1%)	5 (15%)	3 (1.8%)	0 (0%)	2 (2.3%)	939 (3.5%)	
1–3 times/week	115 (28.9%)	43 (30.3%)	39 (27.3%)	33 (29.2%)	6815 (30.4%)	66 (19.2%)	29 (1.7.7%)	22 (23.4%)	15 (17.4%)	4920 (18.1%)	
4–6 times/week	82 (20.6%)	32 (22.5%)	28 (19.6%)	22 (19.5%)	4852 (21.6%)	45 (13.1%)	20 (12.2%)	11 (11.7%)	14 (16.3%)	4966 (18.3%)	
Once a day	117 (29.4%)	42 (29.6%)	42 (29.4%)	33 (29.2%)	6034 (26.8%)	134 (39.1%)	69 (42.1%)	33 (35.1%)	32 (37.2%)	8654 (31.8%)	
>Once a day	67 (18.8%)	22 (15.5%)	26 (18.1%)	19 (16.8%)	3157 (14.1%)	93 (27.1%)	43 (26.2%)	28 (29.8%)	23 (26.8%)	7723 (28.4%)	
Vegetables											28 176 (35.9%)
0–3 times/month	7 (1.8%)	5 (3.5%)	0 (0%)	2 (1.8%)	768 (3.4%)	2 (0.5%)	2 (1.2%)	0 (0%)	0 (0%)	441 (1.6%)	
1–3 times/week	95 (23.9%)	38 (26.8%)	36 (25.2%)	21 (18.6%)	6157 (27.4%)	41 (12%)	17 (10.4%)	15 (16.1%)	9 (10.5%)	4172 (15.3%)	
4–6 times/week	108 (27.1%)	36 (25.4%)	35 (24.5%)	37 (32.7%)	7122 (31.7%)	81 (23.6%)	42 (25.6%)	20 (21.5%)	19 (22.1%)	6792 (25%)	
Once a day	174 (43.7%)	56 (39.4%)	69 (48.2%)	49 (43.4%)	7612 (33.9%)	193 (56.3%)	90 (54.9%)	51 (54.9%)	52 (60.5%)	13 107 (48.2%)	
>Once a day	14 (3.5%)	7 (4.9%)	3 (2.1%)	4 (3.5%)	799 (3.6%)	26 (7.6%)	13 (7.9%)	7 (7.5%)	6 (7%)	2692 (9.9%)	
Milk											13 672 (17.4%)
Never	20 (3.6%)	9 (4.4%)	4 (2.1%)	7 (4.6%)	1138 (4.5%)	57 (7.1%)	26 (7%)	18 (7.4%)	13 (7%)	2836 (7.4%)	
<1 glass/day	44 (8%)	19 (9.3%)	14 (7.2%)	11 (7.3%)	2002 (7.9%)	109 (13.6%)	47 (12.7%)	39 (16%)	23 (12.5%)	4486 (11.8%)	
1–3 glasses/day	348 (63.4%)	130 (63.7%)	123 (63.4%)	95 (62.9%)	15 349 (60.3%)	512 (64.1%)	242 (65.2%)	146 (60.1%)	124 (67%)	24 084 (63.2%)	
>3 glasses/day	137 (25%)	46 (22.6%)	53 (27.3%)	38 (25.2%)	6960 (27.3%)	121 (15.2%)	56 (15.1%)	40 (16.5%)	25 (13.5%)	6704 (17.6%)	
Fish											29 491 (37.5%)
<Once a week	104 (26.6%)	30 (21.4%)	37 (26.4%)	37 (33.3%)	8401 (38.3%)	82 (24.7%)	39 (25.3%)	19 (20.4%)	24 (28.2%)	9975 (37.7%)	
>Once a week	287 (73.4%)	110 (78.6%)	103 (73.6%)	74 (66.7%)	13 532 (61.7%)	250 (75.3%)	115 (74.7%)	74 (79.6%)	61 (71.8%)	16 457 (62.3%)	
Bread											12 377 (15.8%)
White/whitemultigrain/semi‐wholegrain	125 (27.1%)	49 (30.4%)	41 (25%)	35 (25.7%)	8817 (29.8%)	77 (16%)	32 (14.1%)	24 (16.8%)	21 (18.9%)	7389 (20.7%)	
Wholegrain/crispbread	336 (72.9%)	112 (69.6%)	123 (75%)	101 (74.3%)	20 796 (70.2%)	404 (84%)	195 (85.9%)	119 (83.2%)	90 (81.1%)	28 258 (79.3%)	
Red/processed meat^a^											30 455 (38.8%)
0–3 times/month	321 (66%)	104 (61.5%)	122 (70.1%)	95 (66.4%)	13 589 (62.9%)	318 (78.1%)	142 (77.2%)	90 (78.3%)	86 (79.6%)	18 945 (73.9%)	
>Once a week	165 (34%)	65 (38.5%)	52 (29.9%)	48 (33.6%)	8002 (37.1%)	89 (21.9%)	42 (22.8%)	25 (21.7%)	22 (20.4%)	6695 (26.1%)	
Night shift work											22 080 (28.1%)
Yes	49 (12.3%)	8 (5.8%)	20 (14.3%)	21 (17.6%)	5646 (20.8%)	86 (26.6%)	31 (26.7%)	26 (23.9%)	29 (29.6%)	8217 (28.7%)	
No	349 (87.7%)	131 (94.2%)	120 (85.7%)	98 (82.4%)	21 514 (79.2%)	237 (73.4%)	85 (73.3%)	83 (76.1%)	69 (70.4%)	20 401 (71.3%)	
Exercise											9600 (12.2%)
<1 h/times a week	144 (18.2%)	52 (18.1%)	52 (18.4%)	40 (18%)	5078 (16%)	152 (22.1%)	54 (17.6%)	50 (23.4%)	48 (28.7%)	6000 (16.8%)	
>1 h/times a week	648 (81.8%)	236 (81.9%)	230 (81.6%)	182 (82%)	26 653 (84%)	536 (77.9%)	253 (82.4%)	164 (76.6%)	119 (71.3%)	29 768 (83.2%)	
Education											17 109 (21.8%)
Primary school, junior high school	394 (47.5%)	156 (50.5%)	126 (43.6%)	112 (48.5%)	8942 (31.8%)	507 (64%)	251 (68.2%)	143 (59.6%)	113 (61.4%)	12 643 (39.9%)	
Middle school	276 (33.3%)	97 (31.4%)	106 (36.7%)	73 (31.6%)	11 278 (40.1%)	174 (22%)	77 (20.9%)	48 (20%)	49 (26.7%)	9140 (28.8%)	
Senior high school	34 (4.1%)	13 (4.2%)	7 (2.4%)	14 (6%)	2297 (8.1%)	15 (1.9%)	5 (1.4%)	5 (2.1%)	5 (2.7%)	3485 (11%)	
<4 years university college	64 (7.7%)	21 (6.8%)	26 (9%)	17 (7.4%)	3296 (11.7%)	68 (8.6%)	25 (6.8%)	31 (12.9%)	12 (6.5%)	4148 (13.1%)	
>4 years university college	61 (7.4%)	22 (7.1%)	24 (8.3%)	15 (6.5%)	2345 (8.3%)	28 (3.5%)	10 (2.7%)	13 (5.4%)	5 (2.7%)	2275 (7.2%)	

*Note*: Numbers (*n*) and percentages (%) of cases and controls.

Abbreviations: CRC, colorectal cancer; *n*, number.

^a^ Hot dogs/sausages/hamburgers.

### The risk of CRC overall

**FIGURE 2:**
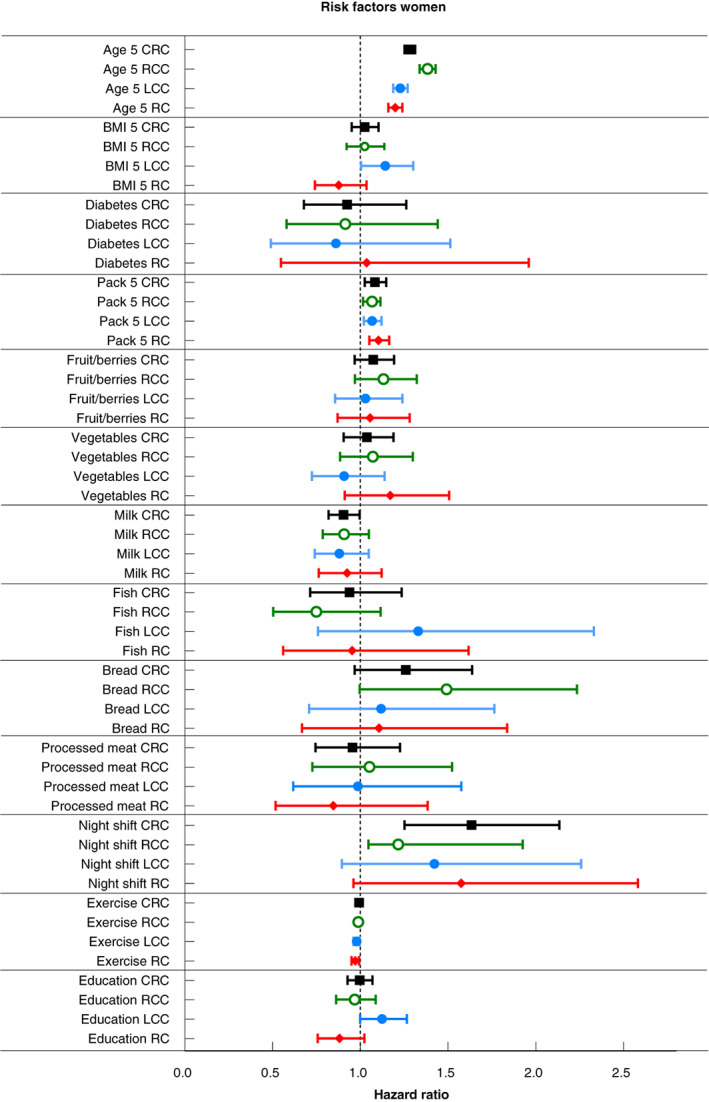
Risk factors for CRC, RCC, LCC and RC for women

**FIGURE 3:**
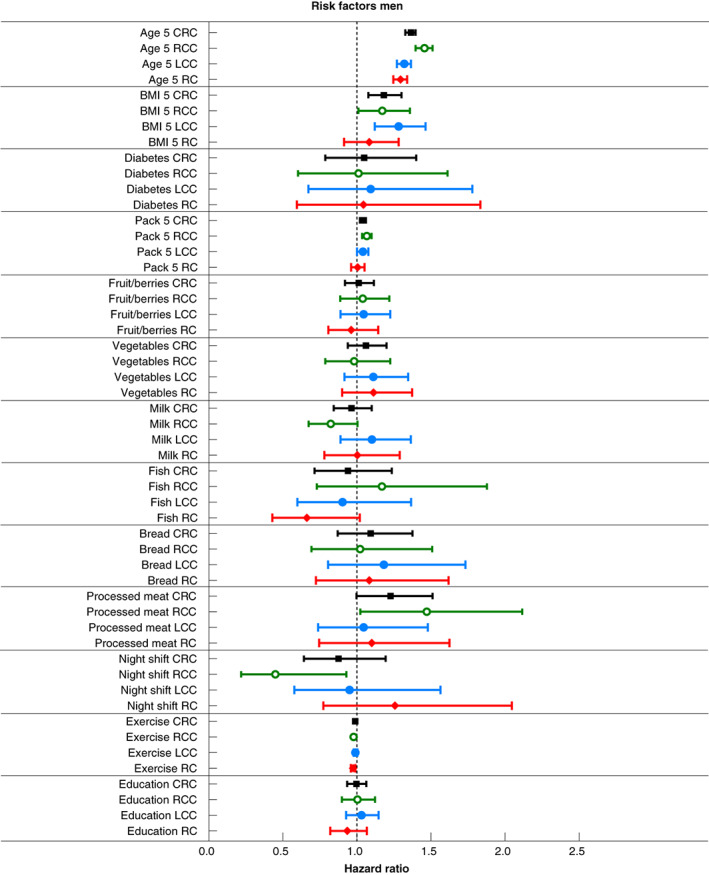
Risk factors for CRC, RCC, LCC and RC for men

Associations between risk factors and CRC, adjusted for age, BMI and smoking, are presented separately for men and women (Figures 2 and 3, Table S2). Older age was associated with an increased risk of CRC in both men [HR 1.36 (1.33–1.40)] and women [HR 1.28 (1.25–1.31)] per 5 years. Higher BMI was significantly associated with an increased risk of CRC in men [HR 1.18(1.08–1.30) per 5 kg/m^2^] but not in women (interaction with sex *p* = 0.02). Smoking was associated with an increased risk of CRC in both men [HR 1.04 (1.02–1.06)] and women [HR 1.08 (1.03–1.15)] per 5 pack years. Diabetes, intake of fruit/berries, vegetables, milk, fish, bread, processed meat and education were not associated with CRC. Night shift work [HR 1.63 (1.25–2.14)] was associated with a higher risk of CRC in women (interaction with sex *p* = 0.002), whereas exercise seemed to be associated with a lower risk of CRC in men [HR 0.99 (0.98–0.99)] although interaction with sex was not significant (*p* = 0.465).

### The risk of CRC by anatomical site

Associations between risk factors and RCC, LCC and RC, adjusted for age, BMI and smoking, are presented separately for men and women (Figures 2 and 3, Table S3). Among men, older age increased the risk of RCC [HR 1.46 (1.40–1.51)], LCC [HR 1.32 (1.27–1.36)] and RC [HR 1.30 (1.25–1.34)] per 5 years, higher BMI increased the risk of LCC [HR 1.28 (1.12–1.46)] per 5 kg/m^2^ and smoking increased the risk of RCC [HR 1.07 (1.04–1.10)] per 5 pack years, whereas exercise reduced the risk of RCC [HR 0.98 (0.96– 0.99)] and RC [HR 0.97 (0.96–0.99)]. Among women, older age [HR 1.38 (1.34–1.43), HR 1.23 (1.19–1.27), HR 1.20 (1.16–1.24) per 5 years] and smoking [HR 1.07 (1.02–1.12), HR 1.07 (1.02–1.12), HR 1.10 (1.05–1.17) per 5 pack years] increased the risk for RCC, LCC and RC, respectively. Night shift work [HR 1.93 (1.22–3.05)] increased the risk of RCC. Analyses of interaction with sex showed significant differences for age in LCC and RC, smoking in RC and night shift work in RCC (*p* < 0.01).

## DISCUSSION

In this population‐based cohort study, candidate CRC risk factors differed by anatomical localization of the tumour and between men and women. The relationship between risk factors and CRC may be more nuanced than previously known. If researchers account for tumour localization and sex in future studies, new insights into pathogenesis, prevention, treatment and follow‐up of CRC might be gained.

### Age

In agreement with previous studies, we found an increased risk of CRC with older age [3, 4, 7]. Through the last four decades there has been ‘a shift to the right’, with increasing incidence of RCC compared with the incidence of LCC and RC [1]. This shift escalates with increasing age and year of diagnosis, and is greater in women than in men [4]. The studied population was not previously screened, hence the median age at colon cancer diagnosis in Norway (73 years for men and 75 years for women) [15] is slightly older than in comparable countries with established screening programmes, for example Finland (median age 71 years for men, 73 years for women) [19].

### Sex

The risk for developing CRC at a specific anatomical site differs for men and women. The risk of developing RC is higher in men, while women more often develop RCC [1]. It has been stated that the lack of reports on sex‐specific estimates of the risk of CRC precludes meta‐analyses and hence evidence for useable cancer prevention guidelines [4]. Many studies including only traditional risk factors such as age, BMI and smoking have demonstrated an increased risk of LCC and RC in men compared with women, and some state that one potential explanation may be unmeasured sex‐specific lifestyle factors [7]. The present study demonstrates different risks for some of these lifestyle factors, with a significant interaction for sex on age, BMI, smoking and night shift work.

### Body mass index

In the present study, a higher BMI increased the risk of LCC in men but not in women or other tumour localizations. In prior studies, higher BMI has been associated with an increased risk of cancer throughout all anatomical subsites, but most of these studies have not analysed the sexes separately [20, 21]. Metabolic syndrome has been shown to increase the risk of CRC in both men and women and is also related to mortality [21]. Consistent with the present study, a previous study has shown that a five‐unit increase in BMI equates to an 18% increased risk of CRC [22].

### Diabetes

Self‐reported diabetes was not associated with CRC in this study. Nonparticipation studies have shown an underestimation of the prevalence of diabetes in HUNT, which could make estimates more modest and associations harder to find [23]. Diabetes has been associated with an increased risk of CRC in many previous studies, particularly with an increased risk of RCC [7, 24]. However, most of these studies did not analyse the genders separately and did not include dietary risk factors or consider the severity of diabetes and its regulation in their analyses.

### Smoking

Smoking was associated with an increased risk of CRC in both men and women, RCC in men and all locations in women (Figures 2 and 3). In previous studies, the association with smoking was strongest for RCC and RC [8]. There was a stronger association in men, but in RC smoking elevates the risk similarly in both men and women [8, 25].

### Diet

As in many previous studies, the present study did not demonstrate any highly significant association with any of the studied dietary factors, either with risk of CRC as a whole or with anatomical subsite of cancer [10]. In the present study, the frequency of intake of sausages/hamburgers was used as a proxy for processed meat. Previous Norwegian studies have found that the association between processed meat and CRC is mainly driven by the intake of sausages [26]. Self‐reported dietary variables vary substantially in quality and validity; hence, the importance of diet in this study may be underestimated [27].

### Night shift work

Night shift work was associated with an increased risk of RCC in women and a significant interaction with sex in our study. Night shift work has previously been associated with a higher risk of CRC [11, 12]. Although night shift work had a significant carcinogenic effect on CRC in America, no such effect was observed in Europe [11]. Some of these studies did not present sex‐specific estimates [12]. Divergent results when examining the association between night shift work and CRC could be because there is no agreement on the definition of ‘night shift work’.

### Exercise

Men who reported higher levels of exercise had a reduced risk of RCC and RC. In several previous studies, physical activity has been shown to reduce risk of CC, whereas associations between physical activity and RC are much less consistent [28]. The frequency and intensity of activity is difficult to quantify, and the amount needed to reduce cancer risk is not known [29, 30]. A systematic review and meta‐analysis found that physical activity was associated with a reduced risk of both RCC and LCC, but RC was not included and no differences were found between men and women [31].

### Education

A substantial proportion of the socioeconomic disparity in risk of CRC is considered attributable to diet and BMI, as measured by educational attainment [32]. This study supports this, as no significant associations were found.

### Factors not analysed

Alcohol consumption has a dose–response relationship with the risk of CRC and drinkers get the disease at an earlier age [8]. In HUNT, the level of self‐reported alcohol consumption was much lower than what was reported in other studies; hence, alcohol consumption was omitted from the present study. Other studies have found that the use of anti‐inflammatory drugs, such as aspirin and nonsteroidal anti‐inflammatory drugs, is associated with a reduced risk of CRC [9]. In Norway, such medications in sufficient doses are prescribed by doctors only to treat other severe diseases (i.e. rheumatic disease) and were therefore not included in our analyses.

Some advocate that factors such as race, sex, age and marital status should be controlled for in all disciplines of research [33]. However, marital status provided no further information in our model and was considered a proxy for other lifestyle factors (data not shown). Ethnicity was not reported in this study. The majority of HUNT participants were Caucasians. Finally, sleep duration has been considered a risk factor for CRC, but data on sleep duration were not available in HUNT [13].

### Strengths and limitations

The population‐based design of the HUNT study, with a large sample size and high participation rate, diminishes the risk of selection bias, provides sufficient study power and reduces the risk of incidental findings [34]. The prospective nature and use of standardized questionnaires limits the potential for recall bias [34]. The risk of differential misclassification of BMI was minimized by the objective and uniform measurement of height and weight by qualified personnel [35]. Another strength of this study is the nearly complete and accurate assessment of the outcome through the high‐quality Cancer Registry of Norway. Furthermore, the use of population controls without CRC diagnosis up to 22 years after registration of the exposure ensures a better separation between cases and controls than in previous studies, where controls are mainly self‐reported healthy or colonoscopy‐negative people.

A limitation is that the questionnaires did not extensively measure the duration or dose of risk factors, hence limiting detailed exploration of each risk factor. Due to limited statistical power, we did not consider the joint effects of risk factors. As this is an observational study, residual confounding cannot be ruled out, and causation cannot be claimed.

The prevalence of CRC in Norway is one of the world's highest and is rising. The representation of different stages in our material agrees with the distribution in Norway overall [15] and other comparable countries [7, 36]. A total of 65 237 (69.5% of those invited) people participated in HUNT2 and 50 807 (54.1% of those invited) participated in HUNT3 [34]. No differences were found between participants and nonparticipants regarding cancer prevalence [23], and only small differences were found in participation between cancer patients and noncancer patients, hence influence in cross‐sectional studies could probably be neglected [37]. Participants in HUNT3 exercised more than nonparticipants [23]. Nonparticipants more often had diabetes, which could lead to an underestimation of the prevalence of diabetes in HUNT [23]. Nonparticipants were found to have higher mortality and lower socio‐economic‐status, which could possibly lead to selection bias [23]. If this is the case, the risk estimates presented will be more modest than if these individuals were included. The nonparticipants were mainly young people in their 20s and people over 80 [23].

## CONCLUSION

In conclusion, our study represents the first large population‐based European cohort study demonstrating that the risk factors for CRC differ by location and between men and women. Further knowledge should be obtained to guide effective prevention policies and possibly reduce CRC disease burden.

## AUTHOR CONTRIBUTIONS

**Siv S. Brenne:** Conceptualization; data curation; formal analysis; funding acquisition; investigation; methodology; visualization; writing – original draft; writing – review and editing. **Eivind Ness‐Jensen:** Conceptualization; methodology; visualization; writing – original draft; writing – review and editing. **Tom‐Harald Edna:** Conceptualization; methodology; visualization; writing – original draft; writing – review and editing. **Stian Lydersen:** Conceptualization; formal analysis; methodology; writing – original draft; writing – review and editing. **Eivor A. Laugsand:** Conceptualization; formal analysis; funding acquisition; investigation; methodology; supervision; visualization; writing – original draft; writing – review and editing.

## CONFLICT OF INTEREST

The authors have no conflicts of interest to declare.

## FUNDING INFORMATION

Research grant from the Central Norway Regional Health Authority.

## ETHICS APPROVAL

Ethical approval for this study was obtained from the Regional Committee for Medical and Health Research Ethics (REC) in Norway.

## PATIENT CONSENT

All HUNT participants gave written informed consent, and it was stated in the information that the collected data could be connected to information from their medical journals as well as other central health registries in Norway.

## PERMISSIONS/DISCLOSURES

The study has used data from the Cancer Registry of Norway. The interpretation and reporting of these data are the sole responsibility of the authors, and no endorsement by the Cancer Registry of Norway is intended nor should be inferred.

## Supporting information


Table S1

Table S2

Table S3

## Data Availability

The data that support the findings of this study are available from HUNT. Restrictions apply to the availability of these data, which were used under licence for this study. Data are available from https://www.ntnu.edu/hunt/research with the permission of HUNT.
