# Supplementary material for: Risk factors for right colon, left colon and rectal cancers differ between men and women: the population‐based HUNT study in Norway
Source: Colorectal Dis. 2022 Sep 13;25(1):44–55. doi: 10.1111/codi.16324 (PMC10087842; doi:10.1111/codi.16324)
Supplement: Supplementary file 1 — Table S1 [file CODI-25-44-s002.docx]

**Supplementary table 1: Known risk factors and their association with CRC, RCC, LCC and RC.**

|  | Risk of CRC | | | Risk RCC | | | Risk LCC | | | Risk RC | | |
| --- | --- | --- | --- | --- | --- | --- | --- | --- | --- | --- | --- | --- |
| Risk factor | Risk | (95%CI) | Ref | Risk | (95%CI) | Ref | Risk | (95%CI) | Ref | Risk | (95%CI) | Ref |
| High age | HR 1.81 | 1.70-1.92 | 1 | OR 1.58 | 1.56-1.59 | 2 | OR 1.34 | 1.33-1.36 | 2 | OR 1.29 | 1.28-1.30 | 2 |
| Male sex | OR 1.4 | ns | 3 | OR 1.15 | 0.97-1.37 | 2 | OR 1.84 | 1.50-2.24 | 2 | OR 2.84 | 2.25-3.58 | 2 |
| Diabetes mellitus | HR 1.26 | 1.18-1.33 | 4 | OR 1.29 | 1.22-1.36 | 2 | OR 1.15 | 1.08-1.22 | 2 | OR 1.12 | 1.06-1.19 | 2 |
| Smoking packyears (≥30) | RR 1.28 | 1.06-1.54 | 5 | HR 1.27 | ns | 1 | HR 1.04 | ns | 1 | HR 1.27 | ns | 1 |
| Alcohol consumption high | RR 1.37 | 1.26-1.49 | 6 | HR 1.05 | 0.75-1.46 | 7 | HR 1.32 | 0.95-1.83 | 7 | RR 1.08 | 1.07-1.10 | 8 |
| BMI, pr 5 kg/m2 | RR 1.06 | 1.04-1.07 | 9 | RR 1.05 | 1.03-1.08 | 9 | RR 1.08 | 1.04-1.11 | 9 | RR 1.02 | 1.01-1.03 | 9 |
| Weight, per 5 kg | RR 1.02 | 1.01-1.02 | 9 | RR 1.02 | 1.00-1.04 | 9 | RR 1.03 | 1.02-1.05 | 9 | RR 1.01 | 1.00-1.02 | 9 |
| Red meat | HR 1.24 | 1.09-1.42 | 10 | RR 1.15 | 0.94-1.41 | 10 | RR 1.29 | 0.88-1.89 | 10 | RR 1.35 | 1.03-1.76 | 10 |
| Processed meat | RR 1.22 | 1.11-1.34 | 11 | RR 1.09 | 0.89-1.33 | 10 | RR 1.10 | 0.86-1.41 | 10 | RR 1.30 | 1.00-1.68 | 10 |
| HRT | RR 0.70 | 0.57-0.85 | 12 | HR 1.17 | 0.67-2.03 | 13 | HR 1.45 | 0.79-2.64 | 13 | HR 0.53 | 0.18-1.56 | 13 |
| Physical activity | RR 0.73 | 0.66-0.81 | 14 | RR 0.73 | 0.66-0.81 | 14 | RR 0.74 | 0.68-0.80 | 14 | OR 0.06 | 0.01-0.35 | 15 |
| Fruit | RR 0.93 | 0.82-1.05 | 16 | RR 0.97 | 0.57-1.64 | 17 | RR 0.91 | 0.53-1.55 | 17 | RR 1.26 | 0.09-18.24 | 16 |
| Vegetables | RR 0.98 | 0.91-1.06 | 16 | RR 0.72 | 0.44-1.20 | 17 | RR 1.13 | 0.66-1.94 | 17 | RR 6.40 | 0.97-42.34 | 16 |
| Fibers (total) | RR 0.90 | 0.86-0.94 | 16 | RR 0.86 | 0.78-0.95 | 18 | RR 0.79 | 0.71-0.87 | 18 | RR 0.80 | 0.56-1.14 | 16 |
| Fish | HR 0.89 | 0.80-0.99 | 8 | HR 0.85 | 0.53-1.37 | 19 | HR 0.70 | 0.44-1.11 | 19 | HR 0.49 | 0.32-0.76 | 19 |
| Milk | RR 0.90 | 0.85-0.94 | 20 | RR 0.76 | 0.45-1.30 | 21 | RR 0.53 | 0.33-0.87 | 21 | RR 0.90 | 0.79-1.02 | 20 |
| Night shift work | OR 1.32 | 1.12-1.55 | 22 | ns | ns | ns | ns | ns | ns | OR 1.32 | 0.81-2.06 | 22 |
| Lower education | IR 1.19 | 1.07-1.31 | 23 | IR 1.07 | 0.93-1.24 | 23 | IR 1.29 | 1.06-1.56 | 23 | IR 1.36 | 1.11-1.65 | 23 |
| Sleep duration long | OR 1.21 | 1.08-1.34 | 24 | HR 1.57 | 0.96-2.56 | 25 | HR 1.01 | 0.56-1.84 | 25 | HR 1.43 | 0.74-2.74 | 25 |

Note: risk estimates of CRC, RCC, LCC and RC as found in the present literature.

Abbreviations: HR = hazard ratio, RR = risk ratio, OR = odds ratio, IR = incidence rate ratio, RaR = random risk, ns = not specified, CRC = colorectal cancer, RCC = Right colon cancer, LCC = Left colon cancer, RC = Rectal cancer, HRT = Hormone replacement therapy, BMI = Body mass index, CI = confidence interval

References

1. Wei, E. K., G. A. Colditz, E. L. Giovannucci, K. Wu, R. J. Glynn, C. S. Fuchs, M. Stampfer, W. Willett, S. Ogino and B. Rosner (2017). "A Comprehensive Model of Colorectal Cancer by Risk Factor Status and Subsite Using Data From the Nurses' Health Study." Am J Epidemiol **185**(3): 224-237.
2. Demb, J., A. Earles, M. E. Martínez, R. Bustamante, A. K. Bryant, J. D. Murphy, L. Liu and S. Gupta (2019). "Risk factors for colorectal cancer significantly vary by anatomic site." BMJ Open Gastroenterol **6**(1): e000313.
3. Oberoi, D. V., M. Jiwa, A. McManus and R. Hodder (2014). "Colorectal cancer--applying a gender lens." Qual Prim Care **22**(2): 71-79.
4. Peeters, P. J., M. T. Bazelier, H. G. Leufkens, F. de Vries and M. L. De Bruin (2015). "The risk of colorectal cancer in patients with type 2 diabetes: associations with treatment stage and obesity." Diabetes Care **38**(3): 495-502.
5. Tsoi, K. K. F., C. Y. Y. Pau, W. K. K. Wu, F. K. L. Chan, S. Griffiths and J. J. Y. Sung (2009). "Cigarette Smoking and the Risk of Colorectal Cancer: A Meta-analysis of Prospective Cohort Studies." Clinical Gastroenterology and Hepatology **7**(6): 682-688.e685.
6. Fagunwa, I. O., M. B. Loughrey and H. G. Coleman (2017). "Alcohol, smoking and the risk of premalignant and malignant colorectal neoplasms." Best Pract Res Clin Gastroenterol **31**(5): 561-568.
7. Bongaerts, B. W., P. A. van den Brandt, R. A. Goldbohm, A. F. de Goeij and M. P. Weijenberg (2008). "Alcohol consumption, type of alcoholic beverage and risk of colorectal cancer at specific subsites." Int J Cancer **123**(10): 2411-2417.
8. Vieira, A. R., L. Abar, D. S. M. Chan, S. Vingeliene, E. Polemiti, C. Stevens, D. Greenwood and T. Norat (2017). "Foods and beverages and colorectal cancer risk: a systematic review and meta-analysis of cohort studies, an update of the evidence of the WCRF-AICR Continuous Update Project." Ann Oncol **28**(8): 1788-1802.
9. Abar, L., A. R. Vieira, D. Aune, J. G. Sobiecki, S. Vingeliene, E. Polemiti, C. Stevens, D. C. Greenwood, D. S. M. Chan, S. Schlesinger and T. Norat (2018). "Height and body fatness and colorectal cancer risk: an update of the WCRF-AICR systematic review of published prospective studies." Eur J Nutr **57**(5): 1701-1720.
10. Cross, A. J., L. M. Ferrucci, A. Risch, B. I. Graubard, M. H. Ward, Y. Park, A. R. Hollenbeck, A. Schatzkin and R. Sinha (2010). "A large prospective study of meat consumption and colorectal cancer risk: an investigation of potential mechanisms underlying this association." Cancer Res **70**(6): 2406-2414.
11. Chan, D. S. M., R. Lau, D. Aune, R. Vieira, D. C. Greenwood, E. Kampman and T. Norat (2011). "Red and Processed Meat and Colorectal Cancer Incidence: Meta-Analysis of Prospective Studies." PLOS ONE **6**(6): e20456.
12. Lin, K. J., W. Y. Cheung, J. Y. Lai and E. L. Giovannucci (2012). "The effect of estrogen vs. combined estrogen-progestogen therapy on the risk of colorectal cancer." Int J Cancer **130**(2): 419-430.
13. Ritenbaugh, C., J. L. Stanford, L. Wu, J. M. Shikany, R. E. Schoen, M. L. Stefanick, V. Taylor, C. Garland, G. Frank, D. Lane, E. Mason, S. G. McNeeley, J. Ascensao, R. T. Chlebowski and I. Women's Health Initiative (2008). "Conjugated equine estrogens and colorectal cancer incidence and survival: the Women's Health Initiative randomized clinical trial." Cancer Epidemiol Biomarkers Prev **17**(10): 2609-2618.
14. Boyle, T., T. Keegel, F. Bull, J. Heyworth and L. Fritschi (2012). "Physical Activity and Risks of Proximal and Distal Colon Cancers: A Systematic Review and Meta-analysis." JNCI: Journal of the National Cancer Institute **104**(20): 1548-1561.
15. Wang, W., Z. Dong, X. Zhang, W. Li, P. Li and X. Chen (2018). "Dietary and the Risk of Sporadic Colorectal Cancer in China: A Case-control Study." Iran J Public Health **47**(9): 1327-1335.
16. Aune, D., D. S. M. Chan, R. Lau, R. Vieira, D. C. Greenwood, E. Kampman and T. Norat (2011). "Dietary fibre, whole grains, and risk of colorectal cancer: systematic review and dose-response meta-analysis of prospective studies." **343**: d6617.
17. Terry, P., E. Giovannucci, K. B. Michels, L. Bergkvist, H. Hansen, L. Holmberg and A. Wolk (2001). "Fruit, vegetables, dietary fiber, and risk of colorectal cancer." J Natl Cancer Inst **93**(7): 525-533.
18. Ma, Y., M. Hu, L. Zhou, S. Ling, Y. Li, B. Kong and P. Huang (2018). "Dietary fiber intake and risks of proximal and distal colon cancers: A meta-analysis." Medicine (Baltimore) **97**(36): e11678.
19. Norat, T., S. Bingham, P. Ferrari, N. Slimani, M. Jenab, M. Mazuir, K. Overvad, A. Olsen, A. Tjønneland, F. Clavel, M. C. Boutron-Ruault, E. Kesse, H. Boeing, M. M. Bergmann, A. Nieters, J. Linseisen, A. Trichopoulou, D. Trichopoulos, Y. Tountas, F. Berrino, D. Palli, S. Panico, R. Tumino, P. Vineis, H. B. Bueno-de-Mesquita, P. H. Peeters, D. Engeset, E. Lund, G. Skeie, E. Ardanaz, C. González, C. Navarro, J. R. Quirós, M. J. Sanchez, G. Berglund, I. Mattisson, G. Hallmans, R. Palmqvist, N. E. Day, K. T. Khaw, T. J. Key, M. San Joaquin, B. Hémon, R. Saracci, R. Kaaks and E. Riboli (2005). "Meat, fish, and colorectal cancer risk: the European Prospective Investigation into cancer and nutrition." J Natl Cancer Inst **97**(12): 906-916.
20. Aune, D., R. Lau, D. S. M. Chan, R. Vieira, D. C. Greenwood, E. Kampman and T. Norat (2012). "Dairy products and colorectal cancer risk: a systematic review and meta-analysis of cohort studies." Ann Oncol **23**(1): 37-45.
21. Larsson, S. C., L. Bergkvist, J. Rutegard, E. Giovannucci and A. Wolk (2006). "Calcium and dairy food intakes are inversely associated with colorectal cancer risk in the Cohort of Swedish Men." Am J Clin Nutr **83**(3): 667-673; quiz 728-669.
22. Wang, X., A. Ji, Y. Zhu, Z. Liang, J. Wu, S. Li, S. Meng, X. Zheng and L. Xie (2015). "A meta-analysis including dose-response relationship between night shift work and the risk of colorectal cancer." Oncotarget **6**(28): 25046-25060.
23. Doubeni, C. A., J. M. Major, A. O. Laiyemo, M. Schootman, A. G. Zauber, A. R. Hollenbeck, R. Sinha and J. Allison (2012). "Contribution of Behavioral Risk Factors and Obesity to Socioeconomic Differences in Colorectal Cancer Incidence." JNCI: Journal of the National Cancer Institute **104**(18): 1353-1362.
24. Chen, Y., F. Tan, L. Wei, X. Li, Z. Lyu, X. Feng, Y. Wen, L. Guo, J. He, M. Dai and N. Li (2018). "Sleep duration and the risk of cancer: a systematic review and meta-analysis including dose–response relationship." BMC Cancer **18**(1): 1149.
25. Zhang, X., E. L. Giovannucci, K. Wu, X. Gao, F. Hu, S. Ogino, E. S. Schernhammer, C. S. Fuchs, S. Redline, W. C. Willett and J. Ma (2013). "Associations of self-reported sleep duration and snoring with colorectal cancer risk in men and women." Sleep **36**(5): 681-688.
