# Supplementary material for: Risk factors for right colon, left colon and rectal cancers differ between men and women: the population‐based HUNT study in Norway
Source: Colorectal Dis. 2022 Sep 13;25(1):44–55. doi: 10.1111/codi.16324 (PMC10087842; doi:10.1111/codi.16324)
Supplement: Supplementary file 2 — Table S2 [file CODI-25-44-s001.docx]

|  |  | **Men** | |  | **Women** | | **Interaction with sex** |
| --- | --- | --- | --- | --- | --- | --- | --- |
|  | **n** | **HR (95% CI)** | **p** | **n** | **HR (95% CI)** | **p** | **p** |
| **Age 5*** | 36593 |  |  | 36593 |  |  |  |
| not adjusted |  | **1.351 (1.319 to 1.383)** | **<0.001** |  | **1.282 (1.258 to 1.313)** | **<0.001** | **0.002** |
| adjusted for BMI, smoking |  | **1.364 (1.326 to 1.396)** | **<0.001** |  | **1.282 (1.252 to 1.313)** | **<0.001** | **<0.001** |
| **BMI 5**** | 31222 |  |  | 36593 |  |  |  |
| adjusted for age |  | **1.211 (1.110 to 1.319)** | **<0.001** |  | 1.030 (0.965 to 1.104) | 0.358 | **0.004** |
| adjusted for age, smoking |  | **1.182 (1.077 to 1.301)** | **<0.001** |  | 1.025 (0.951 to 1.104) | 0.512 | 0.020 |
| **Diabetes** | 31192 |  |  | 36560 |  |  |  |
| adjusted for age |  | 1.099 (0.854 to 1.416) | 0.463 |  | 1.057 (0.812 to 1.375) | 0.681 | 0.832 |
| adjusted for age, BMI, smoking |  | 1.049 (0.786 to 1.400) | 0.748 |  | 0.925 (0.679 to 1.262) | 0.625 | 0.562 |
| **Pack 5***** | 31222 |  |  | 36593 |  |  |  |
| adjusted for age |  | **1.041 (1.020 to 1.067)** | **<0.001** |  | **1.072 (1.041 to 1.104)** | **<0.001** | 0.131 |
| adjusted for age, BMI |  | **1.041 (1.020 to 1.061)** | **<0.001** |  | **1.083 (1.025 to 1.148)** | **0.006** | 0.087 |
| **Fruit/berries** | 22851 |  |  | 27546 |  |  |  |
| adjusted for age |  | 0.981 (0.901 to 1.068) | 0.651 |  | 1.044 (0.951 to 1.146) | 0.368 | 0.332 |
| adjusted for age, BMI, smoking |  | 1.012 (0.919 to 1.114) | 0.815 |  | 1.074 (0.968 to 1.192) | 0.178 | 0.405 |
| **Vegetables** | 22856 |  |  | 27548 |  |  |  |
| adjusted for age |  | 1.033 (0.927 to 1.151) | 0.557 |  | 1.041 (0.922 to 1.176) | 0.516 | 0.923 |
| adjusted for age, BMI, smoking |  | 1.061 (0.938 to 1.200) | 0.350 |  | 1.038 (0.906 to 1.189) | 0.590 | 0.818 |
| **Milk** | 25759 |  |  | 38729 |  |  |  |
| adjusted for age |  | 0.998 (0.888 to 1.122) | 0.977 |  | 0.937 (0.855 to 1.026) | 0.160 | 0.400 |
| adjusted for age, BMI, smoking |  | 0.963 (0.844 to 1.100) | 0.582 |  | 0.904 (0.819 to 0.996) | 0.042 | 0.445 |
| **Fish** | 22324 |  |  | 26764 |  |  |  |
| adjusted for age |  | 0.954 (0.741 to 1.229) | 0.718 |  | 0.954 (0.741 to 1.229) | 0.718 | 0.757 |
| adjusted for age, BMI, smoking |  | 0.939 (0.714 to 1.235) | 0.652 |  | 0.939 (0.714 to 1.235) | 0.652 | 0.669 |
| **Bread** | 30074 |  |  | 36127 |  |  |  |
| adjusted for age |  | 1.103 (0.898 to 1.355) | 0.350 |  | 1.315 (1.030 to 1.679) | 0.028 | 0.281 |
| adjusted for age, BMI, smoking |  | 1.093 (0.869 to 1.375) | 0.447 |  | 1.259 (0.968 to 1.637) | 0.085 | 0.426 |
| **Processed meat****** | 22077 |  |  | 26047 |  |  |  |
| adjusted for age |  | 1.192 (0.987 to 1.440) | 0.068 |  | 0.909 (0.718 to 1.150) | 0.426 | 0.078 |
| adjusted for age, BMI, smoking |  | 1.227 (0.997 to 1.510) | 0.053 |  | 0.956 (0.745 to 1.226) | 0.721 | 0.131 |
| **Night Shift work** | 27558 |  |  | 28939 |  |  |  |
| adjusted for age |  | 0.807 (0.595 to 1.094) | 0.167 |  | **1.605 (1.243 to 2.071)** | **<0.001** | **<0.001** |
| adjusted for age, BMI, smoking |  | 0.875 (0.642 to 1.194) | 0.400 |  | **1.634 (1.251 to 2.135)** | **<0.001** | **0.002** |
| **Exercise** | 32801 |  |  | 36692 |  |  |  |
| adjusted for age |  | **0.986 (0.979 to 0.994)** | **<0.001** |  | **0.986 (0.977 to 0.996)** | **0.007** | 0.992 |
| adjusted for age, BMI, smoking |  | **0.988 (0.980 to 0.996)** | **0.005** |  | 0.993 (0.983 to 1.004) | 0.193 | 0.465 |
| **Education** | 28986 |  |  | 32480 |  |  |  |
| adjusted for age |  | 0.984 (0.928 to 1.044) | 0.601 |  | 0.962 (0.899 to 1.029) | 0.263 | 0.603 |
| adjusted for age, BMI, smoking |  | 0.996 (0.934 to 1.063) | 0.915 |  | 0.996 (0.927 to 1.069) | 0.905 | 0.986 |
|  |  |  |  |  |  |  |  |

Note: Cox regression with colorectal cancer/control as outcome and one risk factor at a time, adjusted for the indicator variable of observational time from HUNT2/3, sex, the interaction term for the risk factor*Sex as well as age, BMI and smoking.

Abbreviations: CRC = colorectal cancer, BMI = body mass index, n= numbers, HR = hazard ratio, 95%CI= 95% confidence interval, p= significance level.

*Per 5 years increase in age, **Per 5 units increase in BMI, ***Per 5 packyears increase in smoking, ****Hot dogs/sausages/hamburgers
