# Supplementary material for: Risk factors for right colon, left colon and rectal cancers differ between men and women: the population‐based HUNT study in Norway
Source: Colorectal Dis. 2022 Sep 13;25(1):44–55. doi: 10.1111/codi.16324 (PMC10087842; doi:10.1111/codi.16324)
Supplement: Supplementary file 3 — Table S3 [file CODI-25-44-s003.docx]

|  |  | **Men** | |  | **Women** | | **Interaction with sex** |
| --- | --- | --- | --- | --- | --- | --- | --- |
|  | **n** | **HR (95% CI)** | **p** | **n** | **HR (95% CI)** | **p** | **p** |
| **Age 5*** | 31222 |  |  | 36594 |  |  |  |
| RCC adj for BMI, smoking |  | **1.456 (1.396 to 1.511)** | **<0.001** |  | **1.383 (1.338 to 1.429)** | **<0.001** | 0.044 |
| LCC adj for BMI, smoking |  | **1.319 (1.270 to 1.364)** | **<0.001** |  | **1.228 (1.188 to 1.270)** | **<0.001** | **0.005** |
| RC adj for BMI, smoking |  | **1.295 (1.246 to 1.338)** | **<0.001** |  | **1.199 (1.159 to 1.240)** | **<0.001** | **0.003** |
| **BMI 5**** | 31222 |  |  | 36594 |  |  |  |
| RCC adj for age, smoking |  | 1.171 (1.010 to 1.357) | 0.035 |  | 1.025 (0.923 to 1.137) | 0.630 | 0.150 |
| LCC adj for age, smoking |  | **1.282 (1.120 to 1.463)** | **<0.001** |  | 1.142 (1.005 to 1.301) | 0.046 | 0.220 |
| RC adj for age, smoking |  | 1.083 (0.913 to 1.282) | 0.370 |  | 0.877 (0.742 to 1.035) | 0.120 | 0.084 |
| **Diabetes** | 31192 |  |  | 36561 |  |  |  |
| RCC adj for age, BMI, smoking |  | 1.011 (0.603 to 1.611) | 0.960 |  | 0.914 (0.580 to 1.441) | 0.700 | 0.760 |
| LCC adj for age, BMI, smoking |  | 1.093 (0.672 to 1.779) | 0.720 |  | 0.861 (0.490 to 1.513) | 0.600 | 0.520 |
| RC adj for age, BMI, smoking |  | 1.044 (0.594 to 1.834) | 0.880 |  | 1.036 (0.548 to 1.960) | 0.910 | 0.990 |
| **Pack 5***** | 31222 |  |  | 36594 |  |  |  |
| RCC adj for age, BMI |  | **1.067 (1.035 to 1.099)** | **<0.001** |  | **1.067 (1.015 to 1.115)** | **0.008** | 0.950 |
| LCC adj for age, BMI |  | 1.041 (1.000 to 1.077) | 0.043 |  | **1.067 (1.020 to 1.120)** | **0.007** | 0.380 |
| RC adj for age, BMI |  | 1.005 (0.961 to 1.051) | 0.820 |  | **1.104 (1.051 to 1.165)** | **<0.001** | **0.006** |
| **Fruit/berries** | 19521 |  |  | 24134 |  |  |  |
| RCC adj for age, BMI, smoking |  | 1.039 (0.887 to 1.219) | 0.630 |  | 1.132 (0.969 to 1.322) | 0.120 | 0.450 |
| LCC adj for age, BMI, smoking |  | 1.045 (0.889 to 1.228) | 0.590 |  | 1.030 (0.856 to 1.240) | 0.750 | 0.910 |
| RC adj for age, BMI, smoking |  | 0.961 (0.807 to 1.144) | 0.660 |  | 1.056 (0.870 to 1.281) | 0.580 | 0.480 |
| **Vegetables** | 19520 |  |  | 24133 |  |  |  |
| RCC adj for age, BMI, smoking |  | 0.981 (0.786 to 1.225) | 0.870 |  | 1.072 (0.885 to 1.299) | 0.480 | 0.560 |
| LCC adj for age, BMI, smoking |  | 1.110 (0.916 to 1.345) | 0.290 |  | 0.908 (0.724 to 1.139) | 0.400 | 0.180 |
| RC adj for age, BMI, smoking |  | 1.112 (0.900 to 1.373) | 0.320 |  | 1.171 (0.911 to 1.506) | 0.220 | 0.760 |
| **Milk** | 21630 |  |  | 34200 |  |  |  |
| RCC adj for age, BMI, smoking |  | 0.823 (0.674 to 1.004) | 0.055 |  | 0.908 (0.786 to 1.049) | 0.190 | 0.430 |
| LCC adj for age, BMI, smoking |  | 1.101 (0.889 to 1.363) | 0.380 |  | 0.880 (0.740 to 1.048) | 0.150 | 0.110 |
| RC adj for age, BMI, smoking |  | 1.003 (0.781 to 1.289) | 0.980 |  | 0.925 (0.763 to 1.122) | 0.430 | 0.620 |
| **Fish** | 19309 |  |  | 23721 |  |  |  |
| RCC adj for age, BMI, smoking |  | 1.169 (0.729 to 1.877) | 0.520 |  | 0.750 (0.504 to 1.116) | 0.160 | 0.160 |
| LCC adj for age, BMI, smoking |  | 0.903 (0.598 to 1.366) | 0.630 |  | 1.330 (0.759 to 2.332) | 0.320 | 0.270 |
| RC adj for age, BMI, smoking |  | 0.662 (0.429 to 1.019) | 0.061 |  | 0.953 (0.561 to 1.617) | 0.860 | 0.280 |
| **Bread** | 25572 |  |  | 31852 |  |  |  |
| RCC adj for age, BMI, smoking |  | 1.022 (0.693 to 1.508) | 0.910 |  | 1.492 (0.997 to 2.235) | 0.052 | 0.190 |
| LCC adj for age, BMI, smoking |  | 1.182 (0.805 to 1.733) | 0.390 |  | 1.118 (0.709 to 1.764) | 0.630 | 0.860 |
| RC adj for age, BMI, smoking |  | 1.083 (0.724 to 1.619) | 0.700 |  | 1.107 (0.667 to 1.837) | 0.690 | 0.950 |
| **Processed meat****** | 19218 |  |  | 23293 |  |  |  |
| RCC adj for age, BMI, smoking |  | 1.471 (1.023 to 2.116) | 0.037 |  | 1.052 (0.727 to 1.524) | 0.790 | 0.210 |
| LCC adj for age, BMI, smoking |  | 1.045 (0.738 to 1.479) | 0.810 |  | 0.986 (0.617 to 1.576) | 0.950 | 0.850 |
| RC adj for age, BMI, smoking |  | 1.100 (0.745 to 1.625) | 0.630 |  | 0.846 (0.517 to 1.384) | 0.500 | 0.410 |
| **Night shift work** | 24405 |  |  | 26113 |  |  |  |
| RCC adj for age, BMI, smoking |  | 0.449 (0.218 to 0.929) | 0.031 |  | **1.925 (1.216 to 3.047)** | **0.005** | **<0.001** |
| LCC adj for age, BMI, smoking |  | 0.950 (0.577 to 1.564) | 0.840 |  | 1.422 (0.895 to 2.259) | 0.140 | 0.230 |
| RC adj for age, BMI, smoking |  | 1.257 (0.773 to 2.046) | 0.360 |  | 1.575 (0.961 to 2.582) | 0.071 | 0.510 |
| **Exercise** | 28300 |  |  | 32625 |  |  |  |
| RCC adj for age, BMI, smoking |  | **0.978 (0.964 to 0.992)** | **0.002** |  | 0.988 (0.973 to 1.003) | 0.130 | 0.310 |
| LCC adj for age, BMI, smoking |  | 0.990 (0.977 to 1.003) | 0.140 |  | 0.979 (0.961 to 0.997) | 0.023 | 0.310 |
| RC adj for age, BMI, smoking |  | **0.974 (0.959 to 0.990)** | **0.001** |  | 0.971 (0.950 to 0.993) | 0.010 | 0.820 |
| **Education** | 24615 |  |  | 28466 |  |  |  |
| RCC adj for age, BMI, smoking |  | 1.004 (0.898 to 1.122) | 0.950 |  | 0.968 (0.862 to 1.087) | 0.580 | 0.640 |
| LCC adj for age, BMI, smoking |  | 1.031 (0.927 to 1.146) | 0.570 |  | 1.124 (0.999 to 1.265) | 0.051 | 0.260 |
| RC adj for age, BMI, smoking |  | 0.935 (0.820 to 1.067) | 0.320 |  | 0.881 (0.757 to 1.024) | 0.098 | 0.530 |

Note: Competing risk survival analysis with right colon, left colon and rectal cancer as separate outcomes and one risk factor at a time, adjusted for the indicator variable of observational time from HUNT2/3, sex, the interaction term for the risk factor*Sex as well as age, BMI and smoking.

Abbreviations: RCC = right colon cancer, LCC = left colon cancer, RC = rectal cancer, BMI = body mass index, n= numbers, HR = hazard ratio, 95%CI= 95% confidence interval, p= significance level.

*Per 5 years increase in age **Per 5 units increase in BMI ***Per 5 packyears increase in smoking, ****Hot dogs/sausages/hamburgers
